# Multivariate Analysis on the Effects of Diabetes and related Clinical Parameters on Cervical Cancer Survival Probability

**DOI:** 10.1038/s41598-018-37694-1

**Published:** 2019-01-31

**Authors:** Syed Wasif Gillani, Hisham A. Zaghloul, Irfan Altaf Ansari, Mohi Iqbal Mohammad Abdul, Syed Azhar Syed Sulaiman, Mirza R. Baig, Hassaan Anwar Rathore

**Affiliations:** 1 Associate Prof. College of Pharmacy, Taibah University, Al-Madinah Al Munawarrah, Saudi Arabia; 2 Associate Prof. Department of Pathology, college of medicine, Taibah University, Al-Madinah Al Munawarrah, Saudi Arabia; 3 Prof. School of Pharmaceutical Sciences, Universiti Sains Malaysia (USM), Penang, Malaysia; 4 Prof. Dubai Pharmacy College, Dubai, UAE; 5 Associate Prof, School of Pharmaceutical Sciences, Universiti Sains Malaysia (USM), Penang, Malaysia

## Abstract

We aimed to evaluate and determine the effect of diabetes mellitus (DM) on overall survival (OS) and cancer-specific survival (CSS) in early stage cervical cancer (CC) patients. Patients with primary cervical cancer and newly diagnosed were selected from ten different cancer specialist hospitals of Malaysia. Patients’ demographic and clinical data were obtained for the prognostic analysis. Kaplan-Meier method was used to estimate patients’ survival time (CSS and OS) with DM status and values were compared using the log-rank test. A total of 19,785 newly diagnosed CC patients were registered during 2010–2016, among them only 16,946 (85.6%) with primary CC tumor. There was no difference in treatment modality between DM and non-DM patients. However intergroup assessment showed that type 2DM have significantly higher rate of mortality in both overall mortality (28.3%) and CC-specific (11.7%) as compared to Type 1DM (17.3%; 5.5%) and non DM patients (12.7%; 9.1%) (*p* < 0.001). Within group assessments showed that Type 2DM patients have better quality of life (mean 7.13 ± 3.67) (*p* < 0.001) and less distress levels (mean 2.41 ± 0.63) (*p* < 0.011) as compared to type1 DM (meant 10.54 ± 2.11; 3.19 ± 1.07). This study concluded that T2DM prognostic effect still remained after adjusting demographic and clinical parameters. Type 1 diabetes mellitus showed better OS and CSS then type2 DM.

## Introduction

Human papillomavirus (HPV -16 & 18) had a persistent link with cervical cancer among women^1^. World Health Organization (WHO) and Global Cancer incidence (GLOBOCAN) estimated that cervical cancer (CC) is ranked 2^nd^ for most common cancer worldwide among women with annual incidence of 5.28 million and mortality 2.66 million each year^1^. About 87% deaths of these cases occurs among women living in developing countries^1^. Women with HIV are at high risk of developing CC^2^, early diagnosis of CC proven to have good prognosis and low rates of mortality^3^. With the help of cytological and molecular screening, early stage diagnosis is possible. In this regard WHO advocates a comprehensive approach to CC prevention & control strategies to identify and deliver effective care^4^.

Malaysia had estimated 4,696 reported cases of CC annually^1^, ministry of health Malaysia stated an average of 2000–3000 hospital admissions of CC annually in the country; and majority of them presenting with late stages of the disease^5^. According to latest Malaysian consensus approximately 2,145 new CC cases are diagnosed annually, it is the 2^nd^ most common cancer among women aged 15–44 years in Malaysia^6^. This report also stated that about 621 CC-specific deaths occur annually in Malaysia. The CC-specific mortality rate in Malaysia is two times higher than Netherlands, United Kingdom and Finland^7^.

The economic burden due to CC is enormous in Malaysia, although surgery and radiotherapy proved to have high cure rates in early stage diagnosis but still several patients die with disease relapse. Several prognostic factors influence the outcome of CC including; lymph node, tumor size, stomach invasion, metastasis^4,6,8^. In recent years, several cohort studies and meta-analysis reported significant influence of diabetes mellitus (DM) on increased risk of cancers with different pathological pathways^9–11^. The underlying mechanism based on hypothesis-involved insulin like growth factor 1 (IGF-1). Hyperglycemia or hyperinsulinemia tends to modulate the over-secretion of free IGF-1, that leads to IGF-1Rs activation and resulting increased proliferation, invasive binding and cellular metastasis^12^.

Identification of IGF-1 at early stage CC reported at preclinical and clinical level but yet studies have not firmly discussed the correlation patterns of CC incidence and DM^13–15^. The pathological and clinical parameters influence on DM, however the effect of diabetes on overall survival (OS) and cervical cancer (CC)-specific (cancer-specific survival – CSS) mortality among early stage CC has not been evaluated so far. Also the concern is focused on the interrelation of diabetes related psychosocial correlates and variability in glycemic index to disease outcome as well as treatment modalities. This hospital-based survival analysis study is the first of its kind; to determine the effect of DM on survival parameters like; OS and CSS among early stage CC patients and also determine the effect to subclasses of DM., type 1 and 2 on the overall mortality and outcome. The psychosocial parameters like, quality of life tool and diabetes distress scale (DDS) were also used to determine the impact on OS and CSS among patients with early stage CC and DM.

## Results

### Patient Characteristics

**Figure 1:**
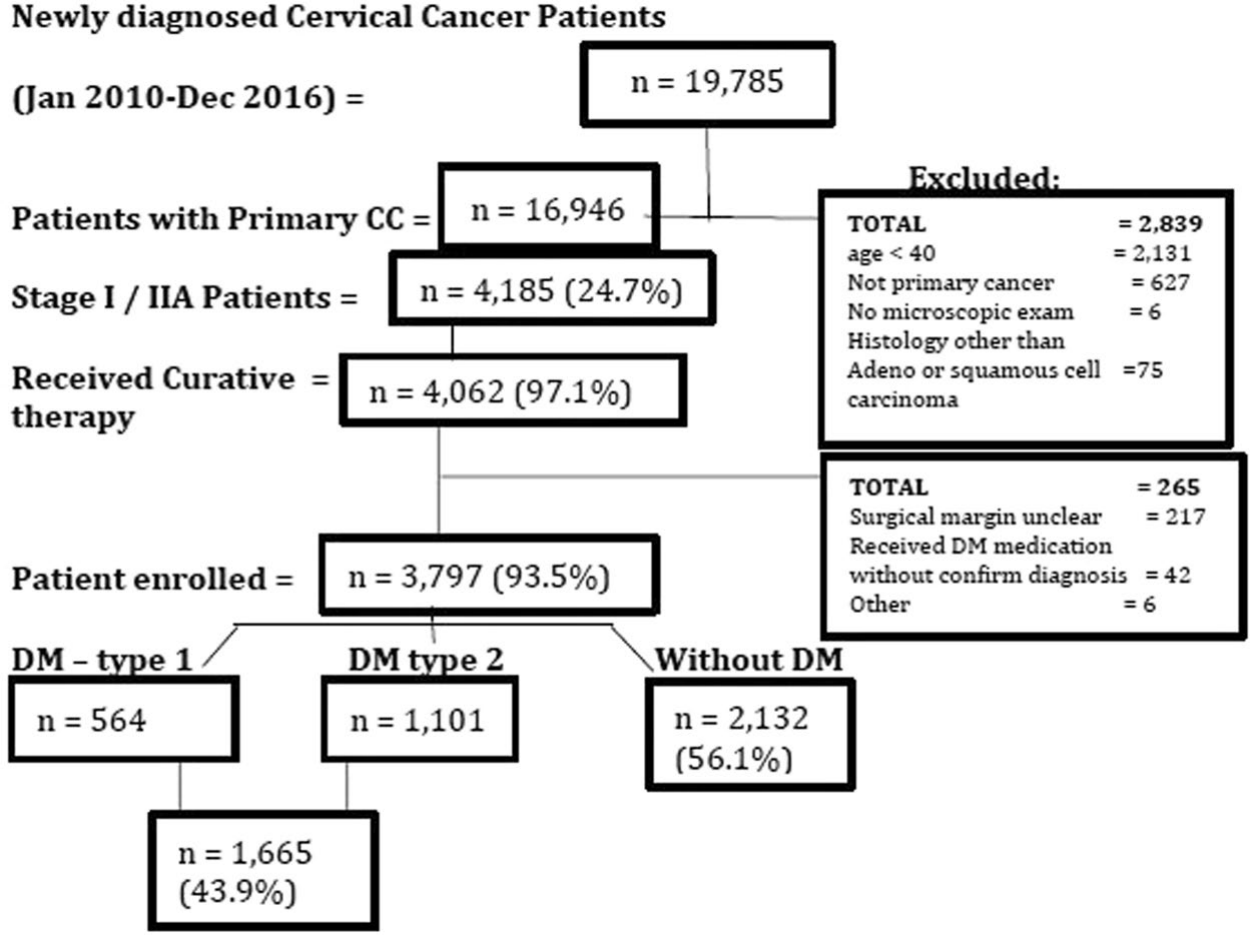
Recruitment and sampling flow chart.

A total of 19,785 newly diagnosed CC patients were registered during 2010–2016, among them only 16,946 (85.6%) with primary CC tumor. After screening for exclusion criteria 3,797 (19.2% out of total registrations) were enrolled in the study (Fig. 1). Subgroups were DM type 1 (n = 564), DM type 2 (n = 1101) and non DM (n = 2,132). Mean age of CC diagnosis among DM type 2 patients was significantly (*p* < 0.001) higher than non-DM patients (Table 1). Also DM type 2 patients (Mean ± SD: 58.0 ± 19.6) have significantly (*p* < 0.001) less follow-up time as compared to type 1 (61.4 ± 22.3) and non-DM patients (75.4 ± 2.9) (Table 1). It was also found that patients with DM (type 1or 2) were significantly probable to develop squamous cell carcinoma (SCC) (*p* < 0.048), high incidences of liver and cardiopulmonary comorbidities (*p* < 0.001), Stage IA/IB (*p* < 0.002) and nodule size ≤6 cm (*p* < 0.021) (Table 1). There was a significant difference (*p* < 0.001) in treatment modality among DM and non-DM patients. Also, intergroup assessment showed that type 2DM have significantly higher rate of mortality in both overall mortality (28.3%) and CC-specific (11.7%) as compared to Type 1DM (17.3%; 5.5%) and non DM patients (12.7%; 9.1%) (*p* < 0.001). Within group assessments showed that Type 2DM patients have better quality of life (mean ± SD: 7.13 ± 3.67) (*p* < 0.001) and less distress levels (mean ± SD: 2.41 ± 0.63) (*p* < 0.011) as compared to type1 DM (mean ± SD: 10.54 ± 2.11; 3.19 ± 1.07).

**Table 1:** Clinical and Diagnostic characteristics of the study population.

Characteristic	Total n (%)	Diabetes mellitus n (%)		Without Diabetes (n%)	p-value
		Type 1 DM	Type 2 DM		
**Study** Patients	3797	564 (14.9)	1101 (29.0)	2132 (56.1)	
**Age** 40–49	875 (23.0)	75 (13.3)	144 (13.1)	656 (30.8)	0.001
50–59	1005 (26.5)	134 (23.8)	381 (34.6)	490 (23.0)	
60–70	1224 (32.2)	207 (36.7)	329 (29.9)	688 (32.3)	
>70	693 (18.3)	148 (26.2)	247 (22.4)	298 (13.9)	
**Age** at diagnosis of DM mean ± SD	—	37.62 ± 5.73	34.25 ± 4.31	—	
**Age** at diagnosis of CC – mean ± SD	57.3 ± 10.1	60.11 ± 9.94	65.2 ± 12.4	53.8 ± 9.87	0.001
**Follow-up time** (months)- mean ± SD	73.1 ± 27.3	61.4 ± 22.3	58.0 ± 19.6	75.4 ± 2.9	0.001
**HbA1C** – mean (SD)	—	8.1 ± 1.67	8.6 ± 2.14	—	0.024
**Stage of CC**					
TIA	1422 (37.4)	110 (19.5)	317 (28.8)	995 (46.7)	0.33
TIB	1317 (34.7)	186 (33.0)	589 (53.5)	542 (25.4)	
TIIA	1058 (27.9)	268 (47.5)	195 (17.7)	595 (27.9)	
TIA/TIB Vs TIIA	—	296 Vs 268	906 Vs 195	—	0.002
**Tumor Size (cm)**					
<4	1367 (36.0)	59 (10.5)	338 (30.7)	970 (45.5)	0.61
4–6	1478 (38.9)	471 (83.5)	605 (55.0)	402 (18.9)	
>6	952 (25.0)	34 (6.0)	158 (14.3)	760 (35.6)	
≤6 cm Vs >6 cm	—	530 Vs 34	943 Vs 158	—	0.021
**Lymph node**					
N0	3568 (94.0)	542 (96.1)	1087 (98.7)	1939 (91.0)	0.23
N1	214 (5.6)	20 (3.5)	14 (1.3)	180 (8.4)	
Unknown	15 (0.4)	2 (0.4)	—	13 (0.6)	
**Histology**					
Squamous cell carcinoma	3211	501	964	1746	0.048
Adenocarcinoma	586	63	137	386	
**EQ-5D-3L** – mean (SD)	—	10.54 ± 2.11	7.13 ± 3.67	—	0.001
**DDS -17** – mean (SD)	—	3.19 ± 1.07	2.41 ± 0.63	—	0.011
**Comorbidities**					
Mild Liver disease	213 (5.6)	23 (4.1)	51 (4.6)	139 (6.5)	0.001
Chronic pulmonary disease	152 (4.0)	62 (11.0)	32 (2.9)	58 (2.7)	0.002
Congestive heart failure	110 (2.9)	18 (3.2)	37 (3.4)	55 (2.6)	0.001
Renal disease	65 (1.7)	23 (4.1)	12 (1.1)	30 (1.4)	0.004
Cerebrovascular disease	38 (1.0)	5 (0.9)	6 (0.5)	27 (1.3)	0.031
Dementia	15 (0.4)	2 (0.4)	—	13 (0.6)	0.84
**Treatment plan**					
Surgery	2539 (66.9)	431 (76.4)	793 (72.0)	1315 (61.7)	0.001
Adjuvant therapy (ć surgery)	817 (21.5)	118 (20.9)	215 (19.5)	484 (22.7)	
Radiotherapy	329 (8.7)	—	84 (7.6)	245 (11.5)	
Chemotherapy (ć radiation)	112 (2.9)	15 (2.7)	9 (0.8)	88 (4.1)	
**Mortality**					
Total	681 (17.9)	98 (17.3)	312 (28.3)	271 (12.7)	0.001
CC- Specific	354 (9.3)	31 (5.5)	129 (11.7)	194 (9.1)	0.001

Significance < 0.05. Age = years, DM- Diabetes Mellitus, CC-Cervical Cancer, HbA1c-glycated hemoglobin, EQ-5D-3L – EuroQOL scale, DDS – 17 – Diabetes distress scale.

### Mortality and Survival Analysis

**Figure 2:**
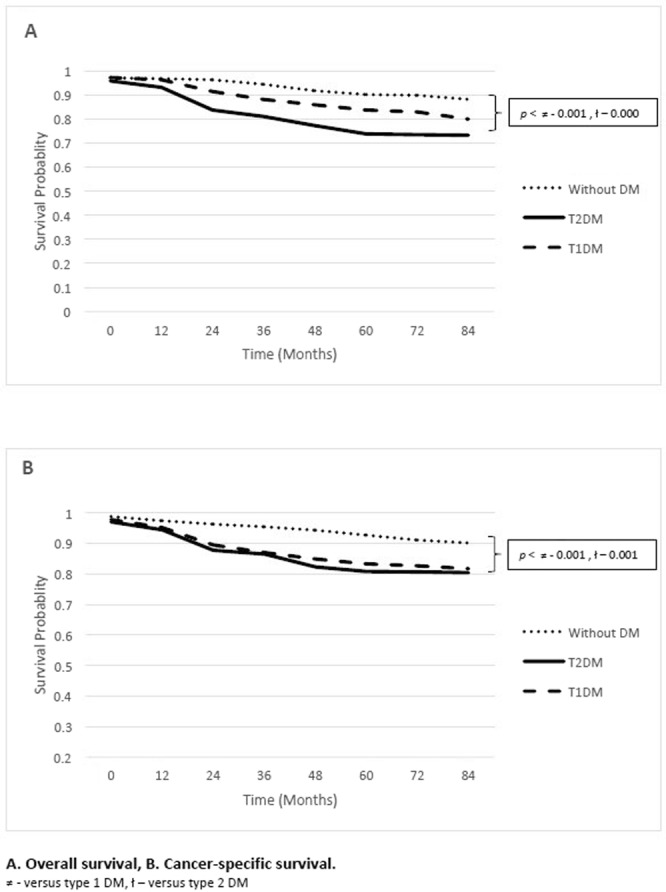
Survival probability of early diagnosed Cervical cancer patients with diabetes subclasses.

During study duration, a total of 681 (17.9%) patients died among them 410 patients were DM (both Type 1 & 2) and rest 271 were non DM group patients. A significant (*p* < 0.0001) difference was reported with shorter OS time among type 2DM as compared to non DM group. Type 1 DM also showed shorter OS time compared to non DM ((*p* < 0.001) but slightly longer than type 2DM (Fig. 2A). Intergroup analysis showed that OS rates of 2 years were 89.1% (DM): 97.3% (non-DM); 79.6% (DM): 90.8% (non-DM) at 5 years and 75.4% (DM): 87.3% (non-DM) at 7 years respectively. Within group analysis showed that type1 DM patients OS rates were significantly higher than type2 DM patients during study follow up time 82.6% versus 71.7% (*p* < 0.001). Similar pattern was found with CC-specific survival analysis (Fig. 2B). However, within group assessment for type1 & 2 DM patients showed no significant difference on CCS rates during study duration.

### Modeling for Probability assessment

Several variables were tested for the probability testing, and many of them showed effect on lower OS and CC-specific survival probability. Therefore, after adjusting age, stage of CC, tumor size, lymph node, comorbidities, histology and treatment modality – DM persisted as potential prognosticator of a lower OS and CSS likelihood (*p* < 0.001) (Fig. 2A,B). Within group assessment showed that type 2 DM patients had lower OS prospect **(**Adjusted HR: 1.61; 95% CI 1.45–2.59; *p* < 0.001) and CSS likelihood (Adjusted HR: 1.56; 95% CI 1.03–2.78; *p* < 0.001) as compared to non DM. Type1 DM patients also reported lower OS value (Adjusted HR: 1.23; 95% CI 1.02–1.81, *p* < 0.001) but CSS probability (Adjusted HR: 1.07; 95% Cl 0.47–1.96, *p* < 0.221) was not different from non DM group patients (Table 2).

**Table 2:** Cox-Regression for interdependent variables in early Cervical Cancer.

Characteristic	Overall Mortality			Cervical-specific		
	Adj HR	95% CI	*p*	Adj HR	95% CI	*p*
**Cohort**						
Without DM	Ref			Ref		
T1DM	1.23	1.02–1.81	0.001	1.07	0.47–1.96	0.221
T2DM	1.61	1.45–2.59	0.001	1.56	1.03–2.78	0.001
**Age** 40–59	Ref			Ref		0.023
50–59	1.05	1.01–1.07	0.024	1.02	1.01–1.13	0.001
60–70	1.83	1.12–3.36	0.001	1.54	1.04–1.97	0.002
>70	1.34	1.00–1.72	0.001	1.17	0.98–1.32	0.001
**Stage of CC**						
TIA	Ref			Ref		
TIB	1.01	0.87–1.03	0.014	1.52	1.32–2.11	0.001
TIIA	1.58	1.27–2.13	0.001	1.86	1.47–2.97	0.001
**Tumor Size (cm)**						
<4	Ref			Ref		
4–6	0.74	0.32–0.97	0.031	0.71	0.64–1.01	0.046
>6	1.42	1.02–1.91	0.001	1.51	1.14–2.03	0.001
**Lymph node**						
N0	Ref			Ref		
N1	4.68	3.27–7.41	0.001	4.99	3.59–8.11	0.001
Unknown	1.99	1.47–2.04	0.022	1.01	0.83–1.29	0.971
**Histology**						
Squamous cell carcinoma	Ref			Ref		
Adenocarcinoma	1.81	1.44–2.37	0.001	2.54	1.79–2.88	0.001
**Comorbidities**						
Mild Liver disease	1.45	0.84–1.85	0.074	0.93	0.54–1.57	0.913
Chronic pulmonary disease	1.17	0.69–1.48	0.541	1.28	0.67–2.01	0.287
Congestive heart failure	1.49	0.70–2.17	0.123	1.41	0.57–2.71	0.436
Renal disease	1.96	1.11–3.49	0.024	0.71	0.33–2.18	0.510
Cerebrovascular disease	1.01	0.72–1.66	0.448	1.48	0.77–2.29	0.391
Dementia	1.43	0.67–3.15	0.415	0.87	0.29–2.79	0.612
**Treatment plan**						
Surgery	Ref			Ref		
Adjuvant therapy (ć surgery)	1.83	1.44–2.69	0.002	1.95	1.33–2.44	0.002
Radiotherapy	2.87	1.94–3.67	0.001	2.54	1.51–3.71	0.001
Chemotherapy (ć radiation)	2.53	1.53–3.01	0.001	2.61	1.69–3.65	0.001

Significance <0.05. Age = years, Adj- Adjusted, HR-Hazard Ratios, CI-Confidence interval, Ref – Reference.

### Heterogeneity and Prognostic influence of Variables on DM

Intergroup analysis showed vital predictive effect of DM on the other variables as; age, stage, tumor size, lymph node, histology and treatment options/modality (Table 3). No significant and strong heterogeneity was found between HR of the subgroups. The ranges for adjusted HR among groups for Overall mortality were 1.023–3.12 and 0.79–3.04 for CC-specific mortality (Table 3).

**Table 3:** Cox-proportional hazards analysis for Diabetes mellitus (with/without) - intergroup assessment.

Characteristic	Patients No	Cervical-specific			Overall mortality		
		Adj HR	95% CI	*p*	Adj HR	95% CI	*p*
**Groups (DM Vs Non-DM)**	3797	1.56	0.79–3.04	0.013	1.87	1.023–3.12	0.001
**Age** 40–59	875	1.17	1.01–2.65	0.004	1.23	1.08–2.89	0.01
50–59	1005	1.74	1.18–3.03	0.001	1.58	0.59–2.17	0.642
60–70	1224	1.94	1.23–3.36	0.001	1.76	0.98–2.99	0.058
>70	693	1.37	0.85–1.98	0.423	1.68	1.24–2.25	0.001
**Stage of CC**							
TIA	1422	1.33	1.15–2.70	0.022	1.31	1.13–2.35	0.001
TIB	1317	1.85	1.10–3.11	0.001	1.88	1.29–2.45	0.001
TIIA	1058	1.01	0.47–2.09	0.64	1.92	0.81–2.41	0.353
**Tumor Size (cm)**							
<4	1367	1.57	0.93–3.13	0.511	1.73	1.13–3.12	0.001
4–6	1478	1.42	0.76–3.47	0.498	1.69	0.91–2.97	0.441
>6	952	1.04	0.47–2.10	0.887	1.32	1.03–2.15	0.032
**Lymph node**							
N0	3568	1.34	0.73–1.91	0.751	1.59	1.07–1.86	0.018
N1	214	1.03	0.10–13.01	0.832	1.57	0.34–18.71	0.754
Unknown	15	2.29	1.07–4.86	0.024	1.82	1.11–3.26	0.001
**Histology**							
Squamous cell carcinoma	3211	3.01	1.47–6.59	0.001	2.79	1.49–4.57	0.001
Adenocarcinoma	586	1.29	0.78–1.93	0.487	1.52	1.17–1.84	0.001
**Treatment plan**							
Surgery	2539	1.84	0.95–3.67	0.324	2.03	1.19–3.32	0.002
Adjuvant therapy (ć surgery)	817	1.75	0.80–3.72	0.455	1.21	0.63–2.22	0.644
Radiotherapy	329	1.21	0.74–2.28	0.61	1.13	1.25–2.57	0.001
Chemotherapy (ć radiation)	112	0.74	0.23–2.19	0.876	1.18	0.69–2.27	0.652

Significance <0.05. Age = years, Adj- Adjusted, HR-Hazard Ratios, CI-Confidence interval, Ref – Reference.

### Diabetes Subclass Heterogeneity Assessment

Within group assessment showed that type2 DM had prognostic influence than type 1DM, on following variables; age, tumor size, low age of diabetes diagnosis, high mean score of QOL, and glycemic index. No strong heterogeneity was reported in the findings; adjusted HR 95% CI range value for CC-specific mortality (0.85–3.34) and overall mortality (OM) (1.33–3.54) were found in the subgroups analysis (Table 4).

**Table 4:** Diabetes Mellitus (type 1 & 2) subgroup analysis of cervical specific survival and overall mortality.

Characteristic	Patients No	Cervical-specific			Overall mortality		
		Adj HR	95% CI	*p*	Adj HR	95% CI	*p*
**Groups (DM Vs Non-DM)**	3797	1.22	0.85–3.34	0.037	2.68	1.33–3.54	0.041
**Age (in years)** 40–59	219	1.23	0.74–2.35	0.446	1.21	1.08–2.78	0.005
50–59	515	1.84	1.01–3.13	0.042	1.76	0.80–2.81	0.456
60–70	536	1.72	0.80–2.46	0.631	1.49	1.24–2.21	0.001
>70	395	1.43	0.85–2.25	0.411	1.17	0.61–1.95	0.889
**Stage of CC**							
TIA	427	1.71	1.03–2.84	0.023	1.74	0.85–2.89	0.512
TIB	775	1.55	0.71–1.94	0.455	1.68	0.80–3.15	0.645
TIIA	463	1.46	0.53–3.29	0.712	1.28	0.71–2.14	0.611
**Tumor Size (cm)**							
<4	397	1.21	1.10–3.21	0.001	1.63	1.12–2.67	0.002
4–6	1076	1.07	0.75–1.49	0.453	1.51	0.81–2.15	0.552
>6	192	1.45	0.95–2.28	0.671	1.39	0.14–2.47	0.865
**Lymph node**							
N0	1629	1.59	1.01–3.48	0.041	1.21	0.09–1.96	0.873
N1	34	1.43	0.85–10.01	0.314	1.01	0.14–8.85	0.891
Unknown	2	0.69	0.13–0.88	0.001	0.49	0.20–1.56	0.91
**Age at Diabetes Diagnosis**							
≤35 years	854	1.75	0.77–2.23	0.332	1.84	1.08–2.44	0.001
>35 years	811	1.72	0.40–2.19	0.764	1.27	0.41–2.29	0.721
**EQ-5d-3L**							
≤7 score	892	1.94	1.13–3.34	0.001	1.76	0.95–2.01	0.084
>7 score	773	1.37	0.94–2.17	0.061	1.43	1.14–2.51	0.001
**HbA1c**							
≤7.5%	618	1.73	1.25–2.86	0.001	1.64	1.12–2.39	0.003
>7.5%	1042	1.09	0.43–2.81	0.713	1.33	1.01–1.84	0.041
**DDS-17**							
2.0–2.9 score	1069	1.23	0.83–1.84	0.613	1.19	0.81–1.62	0.743
≥3.0 score	596	1.09	0.19–4.24	0.77	0.74	0.39–1.34	0.881

Significance <0.05. HbA1c-glycated hemoglobin, EQ-5D-3L – EuroQOL scale, DDS – 17 – Diabetes distress scale.

## Discussion

In this hospital-based case-control study, findings showed that DM patients’ were exhibited adverse prognostic factors for patients with newly and early diagnosis of CC. This finding suggested that type2 DM patients had more mortality rates as compared to type 1, and also the impact on OS probability. According to multivariate analysis; type2 DM showed consistent negative prognostic effect among age (groups), different tumor size, lymph node, histology, age of diabetes diagnosis, glycemic index and treatment modalities. Therefore, type2 DM significantly lower CSS & OS rates and also remained independent predictor for lowering CSS and OS probability.

Literature have reported adverse effect of DM on early stage liver and breast cancers, thus lead to poor prognosis^12,13,16^ and showed increased rates of cancer-specific mortality. This study also reported similar findings with early and newly diagnosed CC patients. In addition to these findings, our study also managed to differentiate the prognostic impact in terms of DM subclasses and related social variables. Past studies reported that CC, breast and livers cancers exhibits high level of IGF-1R overexpression^17,18^. Also IGF-1 levels showed positive association with CC risk has been identified in scientific literature^19^, thus in clinical practice IGF-1R paly vital role to predict the clinical relevance of mortality & disease occurrence at early stages^19,20^.

Patients with either hyperglycemia or hyperinsulinemia might decrease the production of hepatic proteins and binding capacity subsequently increased the concentration of free IGF-1 value in the body^11,12,14,20,21^. Thus concurrent increased IGF-1 levels and activation of IGF axis due to excessive IGF-1R in CC cells may resulted in poor diagnosis^22^. This study also found that more duration of diabetes history significantly and independently lowers both CSS and OS probability (Fig. 2A,B).

The inter-group analysis showed negative impact of DM on OS and CSS, the influence was more significantly prominent among squamous cell carcinoma than adenocarcinoma (Table 3). To the current scientific understanding nearly all types of cancers with positive association to DM were adenocarcinoma histologically^23^. Within group assessment revealed type2 DM more oriented to such effects then type1 DM (Table 1). However, it is still unknown or unclear the effect of different DM subclasses on squamous and adenocarcinoma cells at cellular mechanism of IGF-1 levels and overexpression of IGF-1R axis. More clinical trials are required to determine the systemic pathological pathway of metastasis in adenocarcinoma with hyperglycemia and/or hyperinsulinemia.

Treatment modalities among DM patients were favorable to surgery and radiotherapy alone, other adjuvant therapies showed low OS and CSS (Tables 1 and 3). However, patients with contraindication to surgery exhibit unfavorable risk factors (e.g., inadequate glycemic control), with implied to poor prognosis. A retrospective study reported poor OS rates among obese patients with cervical cancer, and recommended radiotherapy as a primary curative therapy then surgery^17–24^. Also reported that obesity is an independent factor for poor prognosis among cervical cancer patients. Type2 DM patients usually present with overweight /obesity, this could impair the disease outcomes via inadequate treatment dose and/or uncontrolled glycemic index. Sub-therapeutic radiation dose (esp. lateral or oblique treatment)^25^ and others^26^ were contributing barriers in delivering optimum radiation to patients with obesity. Similarly, patients with metastatic lymph nodes and contraindicated to surgery, survival probability is influenced with sub therapeutic levels of either radiotherapy or chemotherapy. This study is limited with body mass index (BMI) values among patients due to missing data in medication profiles, but still morbid obesity is less likely reported in Asian population^27^ so this may slightly influence the study findings. In contrast, patients with unfavorable pathological factors received adjuvant therapy that possibility lead to high risk of disease occurrence and mortality rates.

### Limitations of the study

Several above mentioned clinical variables with potential prognostic characteristics were not analyzed even it might affect the selection of treatment modalities among early and newly diagnosed CC patients with DM.

## Conclusion

This study concluded that diabetes is the potential independent variable for lowering OS and CSS probability among newly diagnosed Cervical cancer patients. Diabetes type 2 mellitus prognostic effect still remained after adjusting demographic and clinical parameters. Type 1 diabetes mellitus showed better OS and CSS than type2 DM. Patient age and duration of diabetes reduced OS and CSS probability in patients with type2 DM then non-diabetes. Hyperglycemic index and poor QOL in patients with DM reported high rates of OS and CSS then non-diabetic patients.

### Practice Implications

This study predicts the prognostic implication of DM for the newly diagnosed early stage CC patient. Findings reported that type 2 DM may increase the risk of cancer relapse and decrease OS even after initiating treatment modalities. Hyperglycemic index and poor QOL in patients with DM reported high rates of OS and CSS then non-diabetic patients. Thus incorporating DM care model in the treatment plan would improve the continuum of care.

## Methodology

### Ethics Approval

The study was performed in compliance with the *WMA* [World Medical Association] *Declaration of Helsinki: Ethical principles for medical research involving human subjects* amended by 59^th^ WMA (number PHRC/HC/11/13), 2013 Seoul, Korea. Study was approved by Clinical Research Committee (CRC), Ministry of Health (MOH) (id: NMRR-10-776-6941), Malaysia. This study protocol followed the Good Clinical Practice (GCP) guidelines, MOH, Malaysia.

### Data Collection

Patients with primary cervical cancer and newly diagnosed between Jan-2010 till Dec-2016 were selected from ten different cancer specialist hospitals of Malaysia. These hospitals located in all the major cities of Malaysia thus covers >90% of total cervical cancer (CC) patients except eastern states i.e., Sabah and Sarawak. Bi-annual follow-up with all the enrolled patients were scheduled throughout the study duration to collect data on clinical and relative parameters. *All the patients were required to sign a mandatory written consent form. Principle investigator ensured the anonymity and privacy of each patient. Patients’ identities were encrypted and all the data was analyzed anonymously*. Informed consent form was also obtained from all the participants at the time of enrollment.

Patient demographic, clinical and pathological status, treatment plans, glycemic index, quality of life relative to diabetes management and comorbidities were obtained for the prognostic analysis. Information and medical records were linked-up with Malaysian death registry to identify and validate the mortalities between 2010 (Jan) and 2017 (May).

### Patients Characteristics and Recruitment

Newly diagnosed primary cervical cancer patients were recruited with inclusion criteria; age >40 years; cervical cancer as primary tumor; stage I-IIA (American Joint Cancer committee –Cancer system (6^th^ edition)^28^ and received curative treatment. Patients were excluded on: cancer history, multiple primary cancers, pathological findings reported other than squamous cell carcinoma or adenocarcinoma, patients with neoadjuvant therapy or unknown treatment modality and either positive or unknown surgical margin.

### Primary and Secondary Endpoints and Definitions

International Classification of Diseases, 9^th^ edition for clinical modifications (ICD-9-CM) codes was used to screen Diabetes mellitus (DM) and other comorbidities in the study population. The diagnosis codes for in-patient and outpatient clinics were included and also Deyo-Charlson comorbidity index were examined^29^. The DM diagnosis either type 1 or type 2 was established if it was reported at least two times in out-patient clinics at different months or at least once in in-patient hospital stay within 1 year. General physician calculated the duration of DM on the basis of first confirmed diagnosis and then counter validated either by patient or caregiver and/or family members. Secondary sources also included ministry of health online repository. Comorbidities were coded and analyzed as dichotomized variable (YES/NO). Followings are the ICD-9-CM codes for comorbidities;

- Type 1 DM (ICD-9-CM code 250.01)
- Type 2 DM (ICD-9-CM code 250.x)
- Mild liver disease (ICD-9-CM codes 571.2,571.4–571.6)
- Chronic Pulmonary Disease (ICD-9-CM codes 416.8, 416.9, 490.x–505.x, 506.4, 508.1, 508.8)
- Congestive heart failure (ICD-9-CM codes 398.91, 402.01, 402.11, 402.91, 404.01, 404.03, 404.11, 404.13, 404.91, 404.93, 425.4–425.9, 428.x)
- Renal disease (ICD-9-CM codes 403.01, 403.11, 403.91, 404.02, 404.03, 404.12, 404.13, 404.92, 404.93, 582.x, 583.0–583.7, 585.x, 586.x, 588.0, V42.0, V45.1, V45.x)
- Cerebrovascular disease (ICD-9-CM codes 362.34, 430.x–438.x)
- Dementia (ICD-9-CM codes 290.x, 294.1, 331.2)

Patients received chemotherapy (CT) or radiotherapy (RT) or combinations within 90 days of surgery were considered as adjuvant therapy/treatment. Participants were observed and scheduled follow-up from the primary diagnosis (CC) to mortality based on Cancer-specific survival (CSS) and death from any cause overall survival (OA) also based on the last follow-up date in the medication profile or hospitals database (i.e., 31^st^ May, 2017).

Follow-up assessments were also included; HbA1c value, Euro quality of life assessment scale (EuroQOL-5D-3L)^30^, Diabetes Distress Scale (DDS-17)^31^. These assessments were made to identify the psychosocial and glycemic variability impact on CC mortality & survival probability. These assessments were collected bi-annually from patients and values were provided in the prognostic analysis.

### Statistical analysis

The baseline values of characteristics were evaluated by using either one-way analysis of variance (continuous variables) or Chi-square test (nominal variables). Kaplan-Meier method was used to estimate patients’ survival time (CSS and OS) with DM status (type 1 & type 2) and values were compared using the log-rank test. The effect of DM (including subclasses) and further potential risk factors were evaluated using Cox proportional hazards model with adjusted Hazard ratios and 95% confidence intervals. All the primary and secondary variables were included in the analysis to determine the effect and consistency of DM (both type 1& type 2) on mortality among study population. A *p* value less than 0.05 (two-sided) was considered statistically significant. Statistical Package for Social Sciences (SPSS ® 22 version) was used to perform analysis.

## Data Availability

Data is available with reasonable request to principle investigator Dr. Syed Wasif Gillani.
